# Phase I and phase II clinical trials in sarcoma: Implications for drug discovery and development

**DOI:** 10.1002/cam4.1958

**Published:** 2019-01-10

**Authors:** Daniel Y. Lee, Arthur P. Staddon, Jacob E. Shabason, Ronnie Sebro

**Affiliations:** ^1^ Department of Radiology University of Pennsylvania Philadelphia Pennsylvania; ^2^ Pennsylvania Hematology Oncology at Pennsylvania Hospital Philadelphia Pennsylvania; ^3^ Department of Radiation Oncology University of Pennsylvania Philadelphia Pennsylvania; ^4^ Department of Orthopedic Surgery University of Pennsylvania Philadelphia Pennsylvania; ^5^ Department of Genetics University of Pennsylvania Philadelphia Pennsylvania; ^6^ Department of Epidemiology and Biostatistics University of Pennsylvania Philadelphia Pennsylvania

**Keywords:** clinical trial

## Abstract

**Background:**

There has been limited progress in the development of novel therapeutics for the treatment of sarcomas. A review of phase I and II clinical trials for sarcomas may give insight into factors influencing sarcoma drug development.

**Methods:**

An exhaustive analysis of phase I and II clinical trials testing drugs for human sarcoma patients between 1 January 2000 and 1 June 2018 was performed using the PubMed search engine, the Thomson Web of Science, and the National Clinical Trials registry. Recorded outcomes included tested drugs, tested histological subtypes, whether the drug was initially developed for sarcoma, reported funding sources, and whether studies led to phase III trials.

**Results:**

Out of 238 studies meeting inclusion criteria, 87% (207 studies) reported funding sources. Of these, 59.9% (124/207) reported industry funding, 52.7% (109/207) reported government funding, and 27.5% (57/207) reported private funding. Only 5% (12/238) of phase I and II trials resulted in phase III trials, with 11 of 12 studies funded by industry. Approximately 90% (214/238) of studies tested drugs that were not initially tested in sarcoma, and 60.1% (143/238) of studies grouped different sarcoma histological subtypes together in the same study.

**Conclusion:**

Industry has funded the majority of phase I and II sarcoma clinical trials that have led to phase III trials. There was a high rate of drugs approved for other cancers and then secondarily tested in sarcoma. Most trials tended to group different sarcoma subtypes rather than studying each subtype separately.

## 1 INTRODUCTION

Sarcomas are rare mesenchymal derived cancers of the bones and soft tissues, with 11 000 incident cases of soft tissue sarcoma (STS) and 3000 incident cases of bone sarcoma occurring each year in the United States.^1^, ^2^ Sarcomas are heterogeneous, with over 60 different known subtypes.^1^, ^2^ Although difficult to generalize, most patients with sarcomas have poor prognoses, with 5 year survival rates between 54% and 80% for patients without metastatic disease^3^, ^4^ and 4%‐27% for patients presenting with metastatic disease.^5^, ^6^

Although other cancers such as lymphoma, melanoma, lung cancer, and breast cancer have undergone treatment revolutions that have resulted in improved patient outcomes over the past two decades,^7^, ^8^, ^9^, ^10^ there have been few advances in the treatment and outcomes of sarcoma patients besides the development of targeted therapies for gastrointestinal stromal tumors (GIST).^11^, ^12^, ^13^

Novel drugs are tested for safety and efficacy in clinical trials prior to use in clinical practice. While many clinical trials for sarcomas have been performed over the past 17 years, a review of the phase I and II clinical trials for sarcomas has the potential to provide great insight into the drug discovery and development process for sarcoma.

We hypothesized that (a) the majority of the funding for phase I and II clinical trials in sarcoma would originate from industry, and (b) the majority of industry‐funded phase I and II sarcoma clinical trials would likely involve drugs already tested in or approved for other cancers. We assessed the source of funding for sarcoma phase I and II trials (government, industry, and private/foundation funding) and the proportion of these clinical trial drugs that were first tested in sarcoma (as a proxy for assessing whether a drug was developed specifically for sarcoma). Finally, we assessed the proportion of phase I and II trials that led to phase III trials.

## 2 METHODS

### 2.1 Data acquisition

An exhaustive search was performed to identify all phase I, II, and III clinical trials primarily involving sarcomas between 1 January 2000 and 1 June 2018, using the PubMed search engine (PubMed.com), the National Clinical Trials registry (www.ClinicalTrials.gov), and Thompson Web of Science. Terms that were searched for in article titles included the following: “sarcoma,” “sarcomas,” “Ewing,” “Ewing's,” “osteosarcoma,” “leiomyosarcoma,” “myxofibrosarcoma,” “liposarcoma,” “synovial sarcoma,” malignant peripheral nerve sheath,” “pleomorphic sarcoma,” and “rhabdomyosarcoma.” Filters used included “Clinical Trial, Phase I,” “Clinical Trial, Phase II,” and “humans.” The phase III trials were excluded, but the phase I/II trial that led to the phase III trial was included. In addition to literature review, National Clinical Trial (NCT) codes were used to further assess whether a clinical trial led to a phase III trial. We collected reported funding sources (industry, government, private), whether the drug was primarily developed for use in sarcoma, number of evaluable patients, tested sarcoma histological subtypes, and whether the clinical trial led to a phase III trial of the tested drug or drug combination for the same indication.

Inclusion criteria for these studies included phase I or phase II clinical trials with titles including the search terms listed above written in English. Exclusion criteria included studies evaluating Kaposi sarcoma, GIST only, radiation therapy, drug schedule optimization for already approved drugs, drug regimen optimization for already approved drugs, clinical trial subanalyses, hyperthermia trials, clinical trial protocol only publications, and studies not looking at phase I or phase II drug clinical trials.

### 2.2 Analysis of funding sources

Clinical trials were sorted by the types of funding sources, including whether the study was funded by a governmental agency (eg, National Cancer Institute (NCI); National Institute of Health (NIH); Ministry of Health, Consumption and Social Welfare of Spain), by industry (eg, Eli Lilly and Company, Amgen, Bristol Myers Squibb, Pfizer), or by a private foundation (ex. Alex's Lemonade Stand Foundation (ALSF), Ewan McGregor, the Sarcoma Foundation of America, Inc). Each trial could have more than one funding source type. Sarcoma phase I and II clinical trials were compared by funding sources and analyzed using two‐sided Fisher's exact tests.

### 2.3 Second‐use drug rate for sarcoma

A drug was considered to have been primarily developed for sarcoma (first‐use) if the first clinical trial using the drug in humans was dedicated to study the drug's effects in the treatment of sarcoma, assessed through retrospective literature review and ClinicalTrials.gov. A drug was considered second‐use if it was initially tested and/or approved for a malignancy other than sarcoma. The proportion of sarcoma phase I and II clinical trials that utilized second‐use drugs were compared by funding source and analyzed using two‐sided Fisher's exact tests.

### 2.4 Other clinical trial characteristics

The average number of patients in sarcoma phase I and II trials was calculated. The proportion of phase I and II clinical trials that grouped different sarcoma histological subtypes together was also calculated. Lastly, trials were grouped by drug class for comparison. In trials testing drug combinations, which always involved an already preapproved (old) drug used in combination with a new drug, the new drug was used for drug class groupings.

## 3 RESULTS

**Figure 1:**
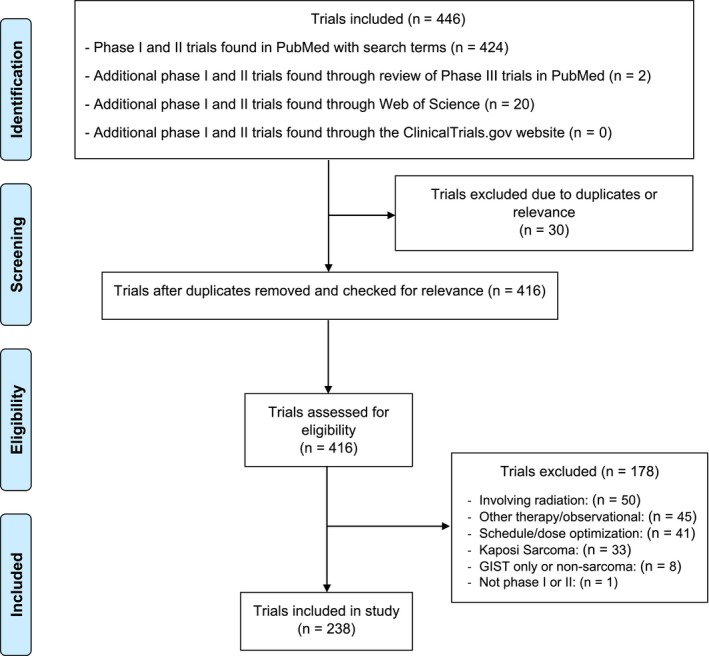
PRISMA diagram demonstrating selection of papers in this review of phase I and phase II sarcoma clinical trials

There were 446 phase I and II trials identified in sarcoma between 1 January 2000 and 1 June 2018. Of these, 208 publications were excluded (duplicates, involving radiation, other therapy, schedule or dose optimization, Kaposi sarcoma, GIST, or not phase I or phase II trials). Overall, 238 publications (53.4%) met the inclusion criteria. Figure 1 shows the study inclusion and exclusion criteria and how the trials were selected. Overall, 69.3% (165/238) of studies were matched with corresponding NCT codes. There were 12 phase I and II clinical trials (5.0%) that resulted in phase III studies of the tested drug or drug combination for the same indication. The list of drugs and drug combinations progressing to phase III clinical trials is given in Tables 1 and 2.

**Table 1:** Drugs evaluated in clinical trials resulting in phase III clinical trials

Drug or drug combination	Patient population	Publication year	Mechanism	FDA status
Gemcitabine + Docetaxel^25^	Newly Diagnosed Ewing sarcoma	2017	Antimetabolite + Microtubule inhibitor	‐
Eribulin^26^	Advanced/Metastatic soft tissue sarcoma (Japanese population)	2017	Microtubule inhibitor	Approved 2016^27^
Olaratumab + doxorubicin^28^	Metastatic soft tissue sarcoma	2016	Antibody + Anthracycline	Approved 2016^20^
Aldoxorubicin^29^	Locally advanced and/or metastatic soft tissue sarcoma	2015	Anthracycline	‐
Evofosfamide + doxorubicin^30^	Advanced unresectable/metastatic soft tissue sarcoma	2014	Alkylating agent prodrug +Anthracycline	‐
Bevacizumab + docetaxel/gemcitabine^31^	Advanced/recurrent soft tissue sarcoma	2012	VEGF‐A Antibody +microtubule inhibitor/antimetabolite	‐
Ridaforolimus^32^	Metastatic/unresectable bone or soft tissue sarcoma	2012	mTOR inhibitor	‐
Eribulin^33^	Progressive or high grade soft tissue sarcoma	2011	Microtubule inhibitor	Approved 2016^27^
Palifosfamide + doxorubicin^34^	Metastatic/unresectable soft tissue sarcoma	2010	Alkylating agent + anthracycline	‐
Trabectedin^35^	Unresectable/metastatic liposarcoma and leiomyosarcoma	2009	Alkylating Agent	Approved 2015^36^
Pazopanib^37^	Advanced soft tissue sarcoma	2009	Tyrosine Kinase inhibitor	Approved 2012^38^
Gemcitabine + Docetaxel^39^	Metastatic soft tissue sarcoma	2007	Antimetabolite + Microtubule inhibitor	‐

**Table 2:** Clinical benefit of clinical trials leading to phase III trials for the same indication

Drug or drug combination	Comparison arm	Clinical Benefit (experimental vs comparison)
Gemcitabine + Docetaxel^25^	None	5 y overall survival: 55% Event free survival: 50%
Eribulin^26^	None	13.2 mo MOS 4.1 mo MPFS
Olaratumab + doxorubicin^28^	Doxorubicin	26.5 mo vs 14.7 mo MOS 6.6 mo vs 4.1 mo MPFS
Aldoxorubicin^29^	Doxorubicin	15.8 mo vs 14.3 mo MOS 5.6 mo vs 2.7 mo MPFS
Evofosfamide + doxorubicin^30^	None	21.5 mo MOS 6.5 mo MPFS
Bevacizumab + docetaxel/gemcitabine^31^	None	31.4% Response rate
Ridaforolimus^32^	None	10 mo MOS 3.8 mo MPFS
Eribulin^33^	None	PFS_12_: 46.9% adipocytic sarcoma, 31.6% leiomyosarcoma 21.1% synovial sarcoma, 19.2% other
Palifosfamide + doxorubicin^34^	Doxorubicin only	7.8 mo vs 4.4 mo MPFS
Trabectedin^35^	Two different schedules	13.9 mo vs 11.8 mo MOS 3.3 mo vs 2.3 mo MPFS
Pazopanib^37^	None	PFS_12_: 44% leiomyosarcoma, 49% synovial sarcoma, 39% other
Gemcitabine + Docetaxel^39^	Gemcitabine	17.9 mo vs 3.0 mo MOS 6.2 mo vs 3.0 mo MPFS

MFS, median overall survival; MPFS, median progression‐free survival; PFS_12_, Progression‐free survival at 12 wk.

### 3.1 Analysis of funding sources

**Figure 2:**
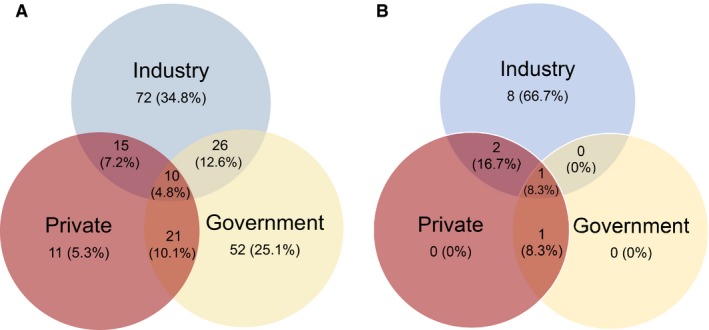
Distribution of funding sources for (A) all clinical trials and (B) studies leading to phase III trials

Two hundred and seven out of 238 phase I and II clinical trials (87.0%) reported funding sources within the published article about the clinical trial (Figure 2). We found that 59.9% (124/207) reported an industry source, 52.7% (109/207) reported a government source, and 27.5% (57/207) reported a private source. The data showed that 34.8% (72/207) of the clinical trials were funded solely by industry, 25.1% (52/207) solely by government sources, and 5.3% (11/207) solely by private sources (Figure 2A). The proportion of industry‐funded studies was higher than government‐funded phase I and II clinical trials, but this was not statistically significant (OR = 1.34, 95% CI (0.89, 2.02), *P* = 0.17). However, the proportion of studies funded solely by industry was statistically greater than the proportion of studies funded solely by government organizations (OR = 1.76, 95% CI (1.16, 2.69), *P* = 0.01). Significantly more studies were funded by industry than by government sources (OR = 3.93, 95% CI (2.55, 6.07), *P* = <0.0001), and significantly more studies were funded by government sources than by private sources (OR = 2.96, 95% CI (1.92, 4.56), *P* = <0.0001).

We found that of all phase I and II clinical trials that resulted in phase III trials, 91.7% (11/12) were funded by industry, 25% (3/12) were funded by government, and 33.3% (4/12) were funded by private organizations (Figure 2B). Additionally, 66.7% (8/12) of these trials were funded solely by industry. The proportion of industry‐funded trials that resulted in phase III trials was significantly higher than those funded by government (OR = 33, 95% CI (2.35, 1572.31), *P* = 0.0028) or private organizations (OR = 22, 95% CI (1.70, 1061.74), *P* = 0.0094). There was no significant difference in the proportion of phase I and phase II clinical trials that resulted in phase III trials that were funded by government versus private organizations (OR = 0.67, 95% CI (0.07, 5.45), *P* = 1.00).

### 3.2 Second‐use drug rate for sarcoma

Approximately 90% (214/238) of phase I and II clinical trials studied drugs that were not initially tested in sarcoma. Trabectedin accounted for 18 of the 24 studies that used first‐use drugs. Drugs that were primarily developed for sarcoma by our criteria included trabectedin (DNA alkylating agent, DNA minor groove binder that inhibits oncogenic FUS‐CHOP transcription factor), brostallicin (DNA alkylating agent), trofosfamide (alkylating agent), mithramycin (RNA synthesis inhibitor), conatumumab (antibody against TRAIL receptor 2), and the SYT‐SSX junction peptide vaccine (specific synovial sarcoma fusion protein vaccine). Trabectedin was the only drug that resulted in a phase III trial. In addition, 91.7% (11/12) of drugs that progressed to phase III trials were considered second‐use by our criteria.

Second‐use drugs were used for approximately 85.5% (106/124) of trials with any industry funding, 93.6% (102/109) of trials with any government funding, and 93.0% (53/57) of clinical trials with any private funding. Government‐funded clinical trials had a higher rate of second‐use drugs than industry‐funded clinical trials, although this was not statistically significant (OR = 2.47, 95% CI (1.01, 6.02), *P* = 0.057). Trials funded solely by industry had a second‐use drug rate of 81.9% (59/72), trials funded solely by government had a second‐use drug rate of 94.2% (49/52), and trials funded solely by private organizations had a second‐use drug rate of 90.9% (10/11).

### 3.3 Other clinical trial characteristics

The majority of phase I and II clinical trials (60.1%, 143/238) grouped different sarcoma subtypes together in the same study. Additionally, 14.7% of trials (35/238) were specifically for bone sarcomas. The average number of patients in the phase I and phase II clinical trials was 45 patients (median 35, standard deviation 39.9).

Table 3 summarizes the analysis from sorting clinical trials by drug class. Kinase inhibitors were the most commonly used drug class, and kinase inhibitors also had significantly more industry funding than other drug classes (OR = 2.38, 95% CI (1.19, 4.77), *P* = 0.014). Other studied drug classes included alkylating agents, monoclonal antibodies/immunotherapeutic agents, anthracyclines, antitumor antibiotics, topoisomerase inhibitors, microtubule inhibitors, antimetabolites, and histone deacetylase inhibitors.

**Table 3:** Summary of studies by drug class

Drug class	# Studies	Industry funding (%)	Gov't funding (%)	Private funding (%)	Second‐use drug (%)	% Studies leading to Phase III
Immunotherapy/Monoclonal Antibodies	40	55.3	52.6	44.7	97.5	5.0 (2)
Alkylating agents	38	73.3	30	23.3	39.5	7.9 (3)
Anthracyclines	13	66.7	16.7	16.7	100	7.7 (1)
Kinase Inhibitors	52	74.5	51.0	21.6	100	3.8 (2)
Microtubule Inhibitors	18	26.7	60	20	100	11.1 (2)
Antimetabolites	28	52.2	69.6	34.8	100	7.1 (2)
Antitumor Antibiotics	2	0	100	0	50	0
Topoisomerase inhibitors	14	45.5	45.5	27.3	100	0
HDAC inhibitors	5	100	60	20	100	0
Other drugs/therapies	28	46.4	60.7	21.4	100	0

## 4 DISCUSSION

The data suggest that most phase I and II clinical trials in sarcoma were funded by industry sources and that industry‐funded drug trials were most likely to lead to phase III clinical trials. Most clinical trials tended to group subtypes of sarcomas. We also found that the vast majority of drugs tested in phase I and II clinical trials were not initially tested in sarcoma, but rather first tried in another malignancy.

There has been limited progress in the development of new drugs for sarcomas. Since the 1970s, cytotoxic chemotherapy with doxorubicin has remained one of the standard first line agents in the treatment of all sarcomas.^11^, ^14^, ^15^ Very few new drugs have been developed, and olaratumab in combination with doxorubicin represents the only new first line therapy for the treatment of advanced soft tissue sarcomas.^11^ There are few novel therapeutic agents for bone sarcomas, with clinical trials over the years focused on multi‐drug combinations of old chemotherapy drugs along with dose and schedule optimization.^14^, ^15^

### 4.1 Analysis of funding sources: industry dominance

With industry supporting the majority of phase I and II studies leading to phase III trials, industry seems to have significantly more influence on sarcoma drug development than government or private organizations, only one government funding source and no private funding sources supporting successful phase I or II clinical trials independently. This is surprising—because sarcomas are so rare, the commercial prospects for industry are limited. Anecdotally, many individuals believe that public funders typically only fund studies where there are few if any commercial prospects. However, the data suggest that industry still remains the major funder for initial studies in sarcoma.

Nevertheless, it is unsurprising to see that a significant proportion of clinical trials were funded by private and government sources. However, the low success rate of clinical trials funded by private and government sources prompts further questions about factors that may be influencing the success of industry‐backed trials.

### 4.2 Second‐use drug rate for sarcoma: the norm rather than the exception

With 90% of studies using second‐use drugs, only a handful of studied drugs were developed primarily for treating sarcoma patients, with trabectedin being the only first‐use drug progressing to phase III trials. Several newly approved drugs for the treatment of sarcomas were first developed for different cancers, such as eribulin mesylate's prior approval for the treatment of metastatic breast cancer.^16^, ^17^ While sarcoma tumor biology may share similarities with other malignancies, there are well‐appreciated differences between the cancer biology of sarcomas and other malignancies that may limit this approach.

Clinical trials funded by industry surprisingly had the lowest rate of second‐use drugs, but this may be due to the large number of industry‐funded studies involving trabectedin. Still, industry provided funding for the largest absolute number of clinical trials testing second‐use drugs. It is presumably easier for pharmaceutical companies to provide already‐available drugs for off‐label testing in sarcoma patients with the hope of broadening the potential drug market. Although it requires pharmaceutical companies relatively little effort to retest existing drugs for sarcoma, it would likely be much more useful to investigate new targeted sarcoma therapies.

### 4.3 Factors and consequences of stagnant drug discovery

The high number of studies grouping different sarcoma histological subtypes (60.1%) together in the same clinical trial may be understandable due to the difficulties in recruiting patients for such a rare disease. However, grouping different types of sarcomas together decreases the power in detecting subtype‐specific treatment responses. There are published reports showing that sarcoma cancer biology varies between sarcoma subtypes, with some drugs such as trabectedin and pazopanib having varying efficacies in different sarcoma subtypes.^11^, ^18^

Many phase III trials did not arise directly from a phase I or II trial for the same indication. For example, a phase III trial for ombrabulin + cisplatin arose from phase I and II studies testing the drug combination in general “solid tumors,” ovarian cancer, and non‐small‐cell lung cancer.^19^ This suggests that indirect evidence is being used in some cases to justify phase III sarcoma studies rather than phase I and II trials.

An additional consequence of the low rate of progression from phase I and II clinical trials for sarcoma drugs is that several drugs were approved by government organizations after only phase II clinical trials. For example, olaratumab with doxorubicin was given an accelerated approval by the FDA for use in patients with soft tissue sarcomas after the phase II trial in 129 patients was published in 2016.^20^ Trabectedin gained approval from the European Medicines Agency (EMA) in 2007 for the treatment of advanced soft tissue sarcoma refractory to anthracyclines and ifosfamide after the phase II study in 270 patients was published in 2009.^15^ Although approved by the EMA for use in general soft tissue sarcomas, this phase II study tested the drug mainly in liposarcoma and leiomyosarcoma patients and lacked a nontrabectedin comparator arm.^15^ The accelerated approval of these drugs instead of the traditional progression through phase III trials prior to approval demonstrates the high demand for new effective sarcoma drugs.

The recent 2016 approval of olaratumab with doxorubicin as a first line treatment for metastatic and unresectable soft tissue sarcomas marks a shift from small molecule inhibitors to biologics in sarcoma treatment.^20^ Going along with this overall shift, there has been an increase in the number of second‐use biologics being tested in sarcoma patients, such as pembrolizumab, bevacizumab, nivolumab, and recently NY‐ESO‐1 T cell immunotherapy.^21^, ^22^, ^23^, ^24^ Although there has been an increased use of second‐use biologics and immunologic therapies, there needs to be a greater focus on developing biologics and immunologic therapies that specifically target sarcoma.

### 4.4 Limitations

This study has some important limitations. First, we only reviewed phase I and II clinical trials published on PubMed, ClinicalTrials.gov, and Thomson Web of Science. Second, the trials included in this study had differing follow‐up times, and it may be difficult to accurately assess the success of more recent trials. Third, our definitions of first‐use and second‐use drugs may not completely be accurate because not all trials are registered on ClinicalTrials.gov and because it is impossible to know the intentions of the individuals designing the drugs and the clinical trials. However, our goal with these definitions was to highlight the obvious dearth of effective targeted therapies being developed for sarcoma and to suggest that there has been very little success with drugs that were not created specifically for sarcoma. Lastly, the exact roles of funding sources were unknown, and we assumed that unreported funding sources were missing completely at random. It was unclear whether a funding source was for an author involved in the study or for the actual study itself—however, we considered these the same.

## 5 CONCLUSION

For the past 17 years, few phase I and II clinical trials for sarcoma drugs have led to phase III trials. The majority of these studies were funded by industry and investigated drugs considered second‐use for sarcoma. Since the use of drugs approved for other indications has not produced strong results, an increase in preclinical work to look for actionable targets makes the most sense. However, the high cost of drug development has led pharmaceutical companies to favor drugs for more common malignancies rather than for sarcomas. Additionally, many sarcoma studies grouped different histological subtypes together since they are so rare, highlighting the difficulty for single centers to run sarcoma clinical trials. However, this may have missed drug‐responsive histological subtypes. Sarcoma subtypes are incredibly important, shown by an increasing number of subtype‐specific therapies, and this may provide a hopeful direction for future drug development.

## CONFLICT OF INTEREST

There are no conflicts of interest.
