# Supplementary material for: Phase I and phase II clinical trials in sarcoma: Implications for drug discovery and development
Source: Cancer Med. 2019 Jan 10;8(2):585–92. doi: 10.1002/cam4.1958 (PMC6382713; doi:10.1002/cam4.1958)
Supplement: Supplementary file 1 [file CAM4-8-585-s001.docx]

**Supplementary Materials**

This document includes the references for all 238 studies that were included in this study in no particular order.

**References**

1. Kawai A, Araki N, Naito Y, et al. Phase 2 study of eribulin in patients with previously treated advanced or metastatic soft tissue sarcoma†. Jpn J Clin Oncol. 2017;47(2):137-144. doi:10.1093/jjco/hyw175
2. Mora J, Castañeda A, Perez-Jaume S, et al. GEIS-21: a multicentric phase II study of intensive chemotherapy including gemcitabine and docetaxel for the treatment of Ewing sarcoma of children and adults: a report from the Spanish sarcoma group (GEIS). Br J Cancer. 2017;117(6):767-774. doi:10.1038/bjc.2017.252
3. Tap WD, Jones RL, Van Tine BA, et al. Olaratumab and doxorubicin versus doxorubicin alone for treatment of soft-tissue sarcoma: an open-label phase 1b and randomised phase 2 trial. The Lancet. 2016;388(10043):488-497. doi:10.1016/S0140-6736(16)30587-6
4. Chawla SP, Papai Z, Mukhametshina G, et al. First-Line Aldoxorubicin vs Doxorubicin in Metastatic or Locally Advanced Unresectable Soft-Tissue Sarcoma: A Phase 2b Randomized Clinical Trial. JAMA Oncol. 2015;1(9):1272. doi:10.1001/jamaoncol.2015.3101
5. Chawla SP, Cranmer LD, Van Tine BA, et al. Phase II Study of the Safety and Antitumor Activity of the Hypoxia-Activated Prodrug TH-302 in Combination With Doxorubicin in Patients With Advanced Soft Tissue Sarcoma. J Clin Oncol. 2014;32(29):3299-3306. doi:10.1200/JCO.2013.54.3660
6. Chawla SP, Staddon AP, Baker LH, et al. Phase II Study of the Mammalian Target of Rapamycin Inhibitor Ridaforolimus in Patients With Advanced Bone and Soft Tissue Sarcomas. J Clin Oncol. 2012;30(1):78-84. doi:10.1200/JCO.2011.35.6329
7. Schöffski P, Ray-Coquard IL, Cioffi A, et al. Activity of eribulin mesylate in patients with soft-tissue sarcoma: a phase 2 study in four independent histological subtypes. Lancet Oncol. 2011;12(11):1045-1052. doi:10.1016/S1470-2045(11)70230-3
8. Demetri GD, Chawla SP, von Mehren M, et al. Efficacy and Safety of Trabectedin in Patients With Advanced or Metastatic Liposarcoma or Leiomyosarcoma After Failure of Prior Anthracyclines and Ifosfamide: Results of a Randomized Phase II Study of Two Different Schedules. J Clin Oncol. 2009;27(25):4188-4196. doi:10.1200/JCO.2008.21.0088
9. Sleijfer S, Ray-Coquard I, Papai Z, et al. Pazopanib, a Multikinase Angiogenesis Inhibitor, in Patients With Relapsed or Refractory Advanced Soft Tissue Sarcoma: A Phase II Study From the European Organisation for Research and Treatment of Cancer–Soft Tissue and Bone Sarcoma Group (EORTC Study 62043). J Clin Oncol. 2009;27(19):3126-3132. doi:10.1200/JCO.2008.21.3223
10. Maki RG, Wathen JK, Patel SR, et al. Randomized Phase II Study of Gemcitabine and Docetaxel Compared With Gemcitabine Alone in Patients With Metastatic Soft Tissue Sarcomas: Results of Sarcoma Alliance for Research Through Collaboration Study 002. J Clin Oncol. 2007;25(19):2755-2763. doi:10.1200/JCO.2006.10.4117
11. Verschraegen CF, Arias-Pulido H, Lee S-J, et al. Phase IB study of the combination of docetaxel, gemcitabine, and bevacizumab in patients with advanced or recurrent soft tissue sarcoma: the Axtell regimen. Ann Oncol. 2012;23(3):785-790. doi:10.1093/annonc/mdr299
12. Verschraegen CF, Chawla SP, Mita MM, et al. A phase II, randomized, controlled trial of palifosfamide plus doxorubicin versus doxorubicin in patients with soft tissue sarcoma (PICASSO). J Clin Oncol. 2010;28(15_suppl):10004-10004. doi:10.1200/jco.2010.28.15_suppl.10004
13. Gelderblom H, Blay JY, Seddon BM, et al. Brostallicin versus doxorubicin as first-line chemotherapy in patients with advanced or metastatic soft tissue sarcoma: An European Organisation for Research and Treatment of Cancer Soft Tissue and Bone Sarcoma Group randomised phase II and pharmacogenetic study. Eur J Cancer. 2014;50(2):388-396. doi:10.1016/j.ejca.2013.10.002
14. Hartmann JT, Oechsle K, Mayer F, Kanz L, Bokemeyer C. Phase II trial of trofosfamide in patients with advanced pretreated soft tissue sarcomas. Anticancer Res. 2003;23(2C):1899-1901.
15. Grohar PJ, Glod J, Peer CJ, et al. A phase I/II trial and pharmacokinetic study of mithramycin in children and adults with refractory Ewing sarcoma and EWS–FLI1 fusion transcript. Cancer Chemother Pharmacol. 2017;80(3):645-652. doi:10.1007/s00280-017-3382-x
16. Demetri GD, Le Cesne A, Chawla SP, et al. First-line treatment of metastatic or locally advanced unresectable soft tissue sarcomas with conatumumab in combination with doxorubicin or doxorubicin alone: A Phase I/II open-label and double-blind study. Eur J Cancer. 2012;48(4):547-563. doi:10.1016/j.ejca.2011.12.008
17. Kawaguchi S, Wada T, Ida K, et al. Phase I vaccination trial of SYT-SSX junction peptide in patients with disseminated synovial sarcoma. J Transl Med. 2005;3:1. doi:10.1186/1479-5876-3-1
18. Schöffski P, Wozniak A, Stacchiotti S, et al. Activity and safety of crizotinib in patients with advanced clear-cell sarcoma with MET alterations: European Organization for Research and Treatment of Cancer phase II trial 90101 ‘CREATE.’ Ann Oncol. 2017;28(12):3000-3008. doi:10.1093/annonc/mdx527
19. Samuels Brian L., Chawla Sant P., Somaiah Neeta, et al. Results of a prospective phase 2 study of pazopanib in patients with advanced intermediate-grade or high-grade liposarcoma. Cancer. 2017;123(23):4640-4647. doi:10.1002/cncr.30926
20. al SV et. Phase Ib/II Study of the Safety and Efficacy of Combination Therapy with Multikinase VEGF Inhibitor Pazopanib and MEK Inhibitor Trametinib In Advan... - PubMed - NCBI. https://proxy.library.upenn.edu:2065/pubmed/28377484. Accessed July 9, 2018.
21. Hyman DM, Sill MW, Lankes HA, et al. A Phase 2 Study of Alisertib (MLN8237) in Recurrent or Persistent Uterine Leiomyosarcoma: An NRG Oncology/Gynecologic Oncology Group Study 0231D. Gynecol Oncol. 2017;144(1):96. doi:10.1016/j.ygyno.2016.10.036
22. Agulnik M, Costa RLB, Milhem M, et al. A phase II study of tivozanib in patients with metastatic and nonresectable soft-tissue sarcomas. Ann Oncol. 2017;28(1):121-127. doi:10.1093/annonc/mdw444
23. Mir O, Brodowicz T, Italiano A, et al. Safety and efficacy of regorafenib in patients with advanced soft tissue sarcoma (REGOSARC): a randomised, double-blind, placebo-controlled, phase 2 trial. Lancet Oncol. 2016;17(12):1732-1742. doi:10.1016/S1470-2045(16)30507-1
24. Schuetze Scott M., Bolejack Vanessa, Choy Edwin, et al. Phase 2 study of dasatinib in patients with alveolar soft part sarcoma, chondrosarcoma, chordoma, epithelioid sarcoma, or solitary fibrous tumor. Cancer. 2016;123(1):90-97. doi:10.1002/cncr.30379
25. Dickson MA, Mahoney MR, Tap WD, et al. Phase II study of MLN8237 (Alisertib) in advanced/metastatic sarcoma. Ann Oncol. 2016;27(10):1855-1860. doi:10.1093/annonc/mdw281
26. Dickson MA, Schwartz GK, Keohan ML, et al. Phase 2 Trial of the CDK4 inhibitor Palbociclib (PD0332991) at 125 mg dose in Well-Differentiated or Dedifferentiated Liposarcoma. JAMA Oncol. 2016;2(7):937-940. doi:10.1001/jamaoncol.2016.0264
27. Schuetze Scott M., Wathen J. Kyle, Lucas David R., et al. SARC009: Phase 2 study of dasatinib in patients with previously treated, high-grade, advanced sarcoma. Cancer. 2015;122(6):868-874. doi:10.1002/cncr.29858
28. Bompas E, Le Cesne A, Tresch-Bruneel E, et al. Sorafenib in patients with locally advanced and metastatic chordomas: a phase II trial of the French Sarcoma Group (GSF/GETO). Ann Oncol. 2015;26(10):2168-2173. doi:10.1093/annonc/mdv300
29. Eroglu Z, Tawbi HA, Hu J, et al. A randomised phase II trial of selumetinib vs selumetinib plus temsirolimus for soft-tissue sarcomas. Br J Cancer. 2015;112(10):1644-1651. doi:10.1038/bjc.2015.126
30. Grignani G, Palmerini E, Ferraresi V, et al. Sorafenib and everolimus for patients with unresectable high-grade osteosarcoma progressing after standard treatment: a non-randomised phase 2 clinical trial. Lancet Oncol. 2015;16(1):98-107. doi:10.1016/S1470-2045(14)71136-2
31. Judson I, Scurr M, Gardner K, et al. Phase II Study of Cediranib in Patients with Advanced Gastrointestinal Stromal Tumors or Soft-Tissue Sarcoma. Clin Cancer Res. 2014;20(13):3603-3612. doi:10.1158/1078-0432.CCR-13-1881
32. Yoo C, Lee J, Rha SY, et al. Multicenter phase II study of everolimus in patients with metastatic or recurrent bone and soft-tissue sarcomas after failure of anthracycline and ifosfamide. Invest New Drugs. 2013;31(6):1602-1608. doi:10.1007/s10637-013-0028-7
33. Valentin T, Fournier C, Penel N, et al. Sorafenib in patients with progressive malignant solitary fibrous tumors: a subgroup analysis from a phase II study of the French Sarcoma Group (GSF/GETO). Invest New Drugs. 2013;31(6):1626-1627. doi:10.1007/s10637-013-0023-z
34. Glade Bender JL, Lee A, Reid JM, et al. Phase I Pharmacokinetic and Pharmacodynamic Study of Pazopanib in Children With Soft Tissue Sarcoma and Other Refractory Solid Tumors: A Children’s Oncology Group Phase I Consortium Report. J Clin Oncol. 2013;31(24):3034-3043. doi:10.1200/JCO.2012.47.0914
35. Martín-Liberal J, López-Pousa A, Broto JM, et al. Phase I trial of sorafenib in combination with ifosfamide in patients with advanced sarcoma: a Spanish group for research on sarcomas (GEIS) study. Invest New Drugs. 2014;32(2):287-294. doi:10.1007/s10637-013-9989-9
36. Kummar S, Allen D, Monks A, et al. Cediranib for Metastatic Alveolar Soft Part Sarcoma. J Clin Oncol. 2013;31(18):2296-2302. doi:10.1200/JCO.2012.47.4288
37. Chevreau C, Cesne AL, Ray‐Coquard I, et al. Sorafenib in patients with progressive epithelioid hemangioendothelioma. Cancer. 119(14):2639-2644. doi:10.1002/cncr.28109
38. Dickson MA, Tap WD, Keohan ML, et al. Phase II Trial of the CDK4 Inhibitor PD0332991 in Patients With Advanced CDK4-Amplified Well-Differentiated or Dedifferentiated Liposarcoma. J Clin Oncol. 2013;31(16):2024-2028. doi:10.1200/JCO.2012.46.5476
39. Santoro A, Comandone A, Basso U, et al. Phase II prospective study with sorafenib in advanced soft tissue sarcomas after anthracycline-based therapy. Ann Oncol. 2013;24(4):1093-1098. doi:10.1093/annonc/mds607
40. Ray-Coquard I, Italiano A, Bompas E, et al. Sorafenib for Patients with Advanced Angiosarcoma: A Phase II Trial from the French Sarcoma Group (GSF/GETO). The Oncologist. 2012;17(2):260-266. doi:10.1634/theoncologist.2011-0237
41. Grignani G, Palmerini E, Dileo P, et al. A phase II trial of sorafenib in relapsed and unresectable high-grade osteosarcoma after failure of standard multimodal therapy: an Italian Sarcoma Group study. Ann Oncol. 2012;23(2):508-516. doi:10.1093/annonc/mdr151
42. Mahmood ST, Agresta S, Vigil C, et al. Phase II Study of Sunitinib Malate, a Multi-Targeted Tyrosine Kinase Inhibitor in Patients with Relapsed or Refractory Soft Tissue Sarcomas. Focus on 3 Prevalent Histologies: Leiomyosarcoma, Liposarcoma, and Malignant Fibrous Histiocytoma. Int J Cancer J Int Cancer. 2011;129(8):1963-1969. doi:10.1002/ijc.25843
43. Sugiura H, Fujiwara Y, Ando M, et al. Multicenter Phase II trial assessing effectiveness of imatinib mesylate on relapsed or refractory KIT-positive or PDGFR-positive sarcoma. J Orthop Sci. 2010;15(5):654-660. doi:10.1007/s00776-010-1506-9
44. Grignani G, Palmerini E, Stacchiotti S, et al. A phase 2 trial of imatinib mesylate in patients with recurrent nonresectable chondrosarcomas expressing platelet-derived growth factor receptor-α or -β: An Italian Sarcoma Group study. Cancer. 2011;117(4):826-831. doi:10.1002/cncr.25632
45. Schöffski P, Blay J-Y, De Greve J, et al. Multicentric parallel phase II trial of the polo-like kinase 1 inhibitor BI 2536 in patients with advanced head and neck cancer, breast cancer, ovarian cancer, soft tissue sarcoma and melanoma. The first protocol of the European Organization for Research and Treatment of Cancer (EORTC) Network Of Core Institutes (NOCI). Eur J Cancer. 2010;46(12):2206-2215. doi:10.1016/j.ejca.2010.03.039
46. Chao J, Budd GT, Chu P, et al. Phase II Clinical Trial of Imatinib Mesylate in Therapy of KIT and/or PDGFRα-expressing Ewing Sarcoma Family of Tumors and Desmoplastic Small Round Cell Tumors. Anticancer Res. 2010;30(2):547-552.
47. Pacey S, Ratain MJ, Flaherty KT, et al. Efficacy and safety of sorafenib in a subset of patients with advanced soft tissue sarcoma from a Phase II randomized discontinuation trial. Invest New Drugs. 2011;29(3):481-488. doi:10.1007/s10637-009-9367-9
48. Hensley ML, Sill MW, Scribner DR, et al. Sunitinib Malate in the treatment of recurrent or persistent uterine leiomyosarcoma: A Gynecologic Oncology Group phase II study. Gynecol Oncol. 2009;115(3):460-465. doi:10.1016/j.ygyno.2009.09.011
49. Maki RG, D’Adamo DR, Keohan ML, et al. Phase II Study of Sorafenib in Patients With Metastatic or Recurrent Sarcomas. J Clin Oncol. 2009;27(19):3133-3140. doi:10.1200/JCO.2008.20.4495
50. Chugh R, Wathen JK, Maki RG, et al. Phase II Multicenter Trial of Imatinib in 10 Histologic Subtypes of Sarcoma Using a Bayesian Hierarchical Statistical Model. J Clin Oncol. 2009;27(19):3148-3153. doi:10.1200/JCO.2008.20.5054
51. George S, Merriam P, Maki RG, et al. Multicenter Phase II Trial of Sunitinib in the Treatment of Nongastrointestinal Stromal Tumor Sarcomas. J Clin Oncol. 2009;27(19):3154-3160. doi:10.1200/JCO.2008.20.9890
52. Bailey HH, Mahoney MR, Ettinger DS, et al. Phase II study of daily oral perifosine in patients with advanced soft tissue sarcoma. Cancer. 107(10):2462-2467. doi:10.1002/cncr.22308
53. Knowling M, Blackstein M, Tozer R, et al. A phase II study of perifosine (D-21226) in patients with previously untreated metastatic or locally advanced soft tissue sarcoma: A National Cancer Institute of Canada Clinical Trials Group trial. Invest New Drugs. 2006;24(5):435-439. doi:10.1007/s10637-006-6406-7
54. Verweij J, van Oosterom A, Blay J-Y, et al. Imatinib mesylate (STI-571 Glivec®, GleevecTM) is an active agent for gastrointestinal stromal tumours, but does not yield responses in other soft-tissue sarcomas that are unselected for a molecular target. Eur J Cancer. 2003;39(14):2006-2011. doi:10.1016/S0959-8049(02)00836-5
55. Kuenen BC, Tabernero J, Baselga J, et al. Efficacy and Toxicity of the Angiogenesis Inhibitor SU5416 As a Single Agent in Patients with Advanced Renal Cell Carcinoma, Melanoma, and Soft Tissue Sarcoma. Clin Cancer Res. 2003;9(5):1648-1655.
56. Brockstein B, Samuels B, Humerickhouse R, Arietta R, al et. Phase II studies of bryostatin-1 in patients with advanced sarcoma and advanced head and neck cancer. Investig New Drugs N Y. 2001;19(3):249-254.
57. Thornton KA, Chen AR, Trucco MM, et al. A Dose Finding Study of Temsirolimus and Liposomal Doxorubicin for Patients with Recurrent and Refractory Bone and Soft Tissue Sarcoma. Int J Cancer J Int Cancer. 2013;133(4). doi:10.1002/ijc.28083
58. Mita MM, Poplin E, Britten CD, et al. Phase I/IIa trial of the mammalian target of rapamycin inhibitor ridaforolimus (AP23573; MK-8669) administered orally in patients with refractory or advanced malignancies and sarcoma. Ann Oncol. 2013;24(4):1104-1111. doi:10.1093/annonc/mds602
59. Schuetze SM, Zhao L, Chugh R, et al. Results of a phase II study of sirolimus and cyclophosphamide in patients with advanced sarcoma. Eur J Cancer. 2012;48(9):1347-1353. doi:10.1016/j.ejca.2012.03.022
60. The cyclin-dependent kinase inhibitor flavopiridol potentiates doxorubicin efficacy in advanced sarcomas: preclinical investigations and results of a phase I dose escalation clinical trial. https://proxy.library.upenn.edu:2065/pmc/articles/PMC3343204/. Accessed July 31, 2018.
61. Geoerger B, Kieran MW, Grupp S, et al. Phase II trial of temsirolimus in children with high-grade glioma, neuroblastoma and rhabdomyosarcoma. Eur J Cancer Oxf Engl 1990. 2012;48(2):253-262. doi:10.1016/j.ejca.2011.09.021
62. Okuno S, Bailey H, Mahoney MR, et al. A Phase 2 Study of Temsirolimus (CCI-779) in Patients With Soft Tissue Sarcomas. Cancer. 2011;117(15):3468-3475. doi:10.1002/cncr.25928
63. Heymach JV. Phase II Study of the Antiangiogenic Agent SU5416 in Patients with Advanced Soft Tissue Sarcomas. Clin Cancer Res. 2004;10(17):5732-5740. doi:10.1158/1078-0432.CCR-04-0157
64. Stacchiotti S, Tamborini E, Lo Vullo S, et al. Phase II study on lapatinib in advanced EGFR-positive chordoma†. Ann Oncol. 2013;24(7):1931-1936. doi:10.1093/annonc/mdt117
65. Stacchiotti S, Longhi A, Ferraresi V, et al. Phase II Study of Imatinib in Advanced Chordoma. J Clin Oncol. 2012;30(9):914-920. doi:10.1200/JCO.2011.35.3656
66. Huh WK, Sill MW, Darcy KM, et al. Efficacy and safety of imatinib mesylate (Gleevec®) and immunohistochemical expression of c-Kit and PDGFR-β in a Gynecologic Oncology Group Phase Il Trial in women with recurrent or persistent carcinosarcomas of the uterus. Gynecol Oncol. 2010;117(2):248-254. doi:10.1016/j.ygyno.2010.01.002
67. von Mehren M, Rankin C, Goldblum JR, et al. Phase II SWOG-directed Intergroup Trial (S0505) of Sorafenib in Advanced Soft Tissue Sarcomas. Cancer. 2012;118(3):770-776. doi:10.1002/cncr.26334
68. Hartmann JT, Mayer F, Schleicher J, et al. Bendamustine hydrochloride in patients with refractory soft tissue sarcoma. Cancer. 110(4):861-866. doi:10.1002/cncr.22846
69. Takahashi M, Takahashi S, Araki N, et al. Efficacy of Trabectedin in Patients with Advanced Translocation‐Related Sarcomas: Pooled Analysis of Two Phase II Studies. The Oncologist. 2017;22(8):979-988. doi:10.1634/theoncologist.2016-0064
70. Martin-Broto J, Pousa AL, de las Peñas R, et al. Randomized Phase II Study of Trabectedin and Doxorubicin Compared With Doxorubicin Alone as First-Line Treatment in Patients With Advanced Soft Tissue Sarcomas: A Spanish Group for Research on Sarcoma Study. J Clin Oncol. 2016;34(19):2294-2302. doi:10.1200/JCO.2015.65.3329
71. Bui-Nguyen B, Butrynski JE, Penel N, et al. A phase IIb multicentre study comparing the efficacy of trabectedin to doxorubicin in patients with advanced or metastatic untreated soft tissue sarcoma: The TRUSTS trial. Eur J Cancer. 2015;51(10):1312-1320. doi:10.1016/j.ejca.2015.03.023
72. Kawai A, Araki N, Sugiura H, et al. Trabectedin monotherapy after standard chemotherapy versus best supportive care in patients with advanced, translocation-related sarcoma: a randomised, open-label, phase 2 study. Lancet Oncol. 2015;16(4):406-416. doi:10.1016/S1470-2045(15)70098-7
73. Pautier P, Floquet A, Chevreau C, et al. Trabectedin in combination with doxorubicin for first-line treatment of advanced uterine or soft-tissue leiomyosarcoma (LMS-02): a non-randomised, multicentre, phase 2 trial. Lancet Oncol. 2015;16(4):457-464. doi:10.1016/S1470-2045(15)70070-7
74. Kasper B, Reichardt P, Pink D, et al. Combination of Trabectedin and Gemcitabine for Advanced Soft Tissue Sarcomas: Results of a Phase I Dose Escalating Trial of the German Interdisciplinary Sarcoma Group (GISG). Mar Drugs. 2015;13(1):379-388. doi:10.3390/md13010379
75. A phase II study to determine the efficacy and safety of oral treosulfan in patients with advanced pre‐treated Ewing sarcoma ISRCTN11631773 - Michelagnoli - 2015 - Pediatric Blood &amp; Cancer - Wiley Online Library. https://proxy.library.upenn.edu:2894/doi/full/10.1002/pbc.25156. Accessed July 10, 2018.
76. Baruchel S, Pappo A, Krailo M, et al. A phase 2 trial of trabectedin in children with recurrent rhabdomyosarcoma, Ewing sarcoma and non-rhabdomyosarcoma soft tissue sarcomas: A report from the Children’s Oncology Group. Eur J Cancer. 2012;48(4):579-585. doi:10.1016/j.ejca.2011.09.027
77. Monk BJ, Blessing JA, Street DG, Muller CY, Burke JJ, Hensley ML. A PHASE II EVALUATION OF TRABECTEDIN IN THE TREATMENT OF ADVANCED, PERSISTENT, OR RECURRENT UTERINE LEIOMYOSARCOMA: A GYNECOLOGIC ONCOLOGY GROUP STUDY. Gynecol Oncol. 2012;124(1):48-52. doi:10.1016/j.ygyno.2011.09.019
78. Gronchi A, Bui BN, Bonvalot S, et al. Phase II clinical trial of neoadjuvant trabectedin in patients with advanced localized myxoid liposarcoma. Ann Oncol. 2012;23(3):771-776. doi:10.1093/annonc/mdr265
79. Paz-Ares L, López-Pousa A, Poveda A, et al. Trabectedin in pre-treated patients with advanced or metastatic soft tissue sarcoma: a phase II study evaluating co-treatment with dexamethasone. Invest New Drugs. 2012;30(2):729-740. doi:10.1007/s10637-010-9561-9
80. Sessa C, Perotti A, Noberasco C, et al. Phase I clinical and pharmacokinetic study of trabectedin and doxorubicin in advanced soft tissue sarcoma and breast cancer. Eur J Cancer. 2009;45(7):1153-1161. doi:10.1016/j.ejca.2008.11.019
81. Blay J-Y, Mehren M von, Samuels BL, et al. A Phase I Combination Study of Trabectedin and Doxorubicin in Patients With Soft Tissue Sarcoma. Clin Cancer Res Off J Am Assoc Cancer Res. 2008;14(20):6656-6662. doi:10.1158/1078-0432.CCR-08-0336
82. Leahy M, Ray-Coquard I, Verweij J, et al. Brostallicin, an agent with potential activity in metastatic soft tissue sarcoma: A phase II study from the European Organisation for Research and Treatment of Cancer Soft Tissue and Bone Sarcoma Group. Eur J Cancer. 2007;43(2):308-315. doi:10.1016/j.ejca.2006.09.014
83. Long HJ, Blessing JA, Sorosky J. Phase II trial of dacarbazine, mitomycin, doxorubicin, and cisplatin with sargramostim in uterine leiomyosarcoma: A Gynecologic Oncology Group study. Gynecol Oncol. 2005;99(2):339-342. doi:10.1016/j.ygyno.2005.06.002
84. Siehl JM, Thiel E, Schmittel A, et al. Ifosfamide/liposomal daunorubicin is a well tolerated and active first-line chemotherapy regimen in advanced soft tissue sarcoma. Cancer. 104(3):611-617. doi:10.1002/cncr.21211
85. Le Cesne A, Blay JY, Judson I, et al. Phase II Study of ET-743 in Advanced Soft Tissue Sarcomas: A European Organisation for the Research and Treatment of Cancer (EORTC) Soft Tissue and Bone Sarcoma Group Trial. J Clin Oncol. 2005;23(3):576-584. doi:10.1200/JCO.2005.01.180
86. Winkle PV, Angiolillo A, Krailo M, et al. Ifosfamide, carboplatin, and etoposide (ICE) reinduction chemotherapy in a large cohort of children and adolescents with recurrent/refractory sarcoma: The Children’s Cancer Group (CCG) experience. Pediatr Blood Cancer. 44(4):338-347. doi:10.1002/pbc.20227
87. Patel SR, Papadopolous N, Raymond AK, et al. A phase II study of cisplatin, doxorubicin, and ifosfamide with peripheral blood stem cell support in patients with skeletal osteosarcoma and variant bone tumors with a poor prognosis. Cancer. 101(1):156-163. doi:10.1002/cncr.20317
88. Yovine A, Riofrio M, Blay JY, et al. Phase II Study of Ecteinascidin-743 in Advanced Pretreated Soft Tissue Sarcoma Patients. J Clin Oncol. 2004;22(5):890-899. doi:10.1200/JCO.2004.05.210
89. Trent JC, Beach J, Burgess MA, et al. A two-arm phase II study of temozolomide in patients with advanced gastrointestinal stromal tumors and other soft tissue sarcomas. Cancer. 98(12):2693-2699. doi:10.1002/cncr.11875
90. Talbot SM, Keohan ML, Hesdorffer M, et al. A phase II trial of temozolomide in patients with unresectable or metastatic soft tissue sarcoma. Cancer. 98(9):1942-1946. doi:10.1002/cncr.11730
91. Laverdiere C, Kolb EA, Supko JG, et al. Phase II study of ecteinascidin 743 in heavily pretreated patients with recurrent osteosarcoma. Cancer. 98(4):832-840. doi:10.1002/cncr.11563
92. Sandler E, Lyden E, Ruymann F, et al. Efficacy of ifosfamide and doxorubicin given as a phase II “window” in children with newly diagnosed metastatic rhabdomyosarcoma: A report from the Intergroup Rhabdomyosarcoma Study Group*. Med Pediatr Oncol. 37(5):442-448. doi:10.1002/mpo.1227
93. Ecteinascidin-743: A Marine-Derived Compound in Advanced, Pretreated Sarcoma Patients—Preliminary Evidence of Activity: Journal of Clinical Oncology: Vol 19, No 5. http://proxy.library.upenn.edu:2779/doi/abs/10.1200/jco.2001.19.5.1248?trendmd-shared=0. Accessed August 11, 2018.
94. Comandone A, Bretti S, Bertetto O, Oliva C, Bergnolo P, Bumma C. Low dose adriamycin and ifosfamide in the treatment of advanced adult soft tissue sarcomas. Anticancer Res. 2000;20(3B):2077-2080.
95. Hawkins D, Barnett T, Bensinger W, Gooley T, Sanders J. Busulfan, melphalan, and thiotepa with or without total marrow irradiation with hematopoietic stem cell rescue for poor-risk Ewing-sarcoma-family tumors. Med Pediatr Oncol. 34(5):328-337. doi:10.1002/(SICI)1096-911X(200005)34:5<328::AID-MPO3>3.0.CO;2-4
96. Chisholm JC, Machin D, McDowell H, et al. Efficacy of carboplatin given in a phase II window study to children and adolescents with newly diagnosed metastatic soft tissue sarcoma. Eur J Cancer. 2007;43(17):2537-2544. doi:10.1016/j.ejca.2007.08.024
97. Garcia-Carbonero R, Supko JG, Maki RG, et al. Ecteinascidin-743 (ET-743) for Chemotherapy-Naive Patients With Advanced Soft Tissue Sarcomas: Multicenter Phase II and Pharmacokinetic Study. J Clin Oncol. 2005;23(24):5484-5492. doi:10.1200/JCO.2005.05.028
98. Merimsky O, Meller I, Flusser G, et al. Gemcitabine in soft tissue or bone sarcoma resistant to standard chemotherapy: a phase II study. Cancer Chemother Pharmacol. 2000;45(2):177-181. doi:10.1007/s002800050027
99. Basaran M, Bavbek ES, Saglam S, et al. A Phase II Study of Cisplatin, Ifosfamide and Epirubicin Combination Chemotherapy in Adults with Nonmetastatic and Extremity Osteosarcomas. Oncology. 2007;72(3-4):255-260. doi:10.1159/000113017
100. van Rijswijk REN, Vermorken JB, Reed N, et al. Cisplatin, doxorubicin and ifosfamide in carcinosarcoma of the female genital tract. A phase II study of the European Organization for Research and Treatment of Cancer Gynaecological Cancer Group (EORTC 55923). Eur J Cancer. 2003;39(4):481-487. doi:10.1016/S0959-8049(02)00740-2
101. Tawbi HA, Burgess M, Bolejack V, et al. Pembrolizumab in advanced soft-tissue sarcoma and bone sarcoma (SARC028): a multicentre, two-cohort, single-arm, open-label, phase 2 trial. Lancet Oncol. 2017;18(11):1493-1501. doi:10.1016/S1470-2045(17)30624-1
102. Ben-Ami Eytan, Barysauskas Constance M., Solomon Sarah, et al. Immunotherapy with single agent nivolumab for advanced leiomyosarcoma of the uterus: Results of a phase 2 study. Cancer. 2017;123(17):3285-3290. doi:10.1002/cncr.30738
103. Chisholm JC, Merks JHM, Casanova M, et al. Open-label, multicentre, randomised, phase II study of the EpSSG and the ITCC evaluating the addition of bevacizumab to chemotherapy in childhood and adolescent patients with metastatic soft tissue sarcoma (the BERNIE study). Eur J Cancer. 2017;83:177-184. doi:10.1016/j.ejca.2017.06.015
104. Navid Fariba, Santana Victor M., Neel Michael, et al. A phase II trial evaluating the feasibility of adding bevacizumab to standard osteosarcoma therapy. Int J Cancer. 2017;141(7):1469-1477. doi:10.1002/ijc.30841
105. Anderson PM, Bielack SS, Gorlick RG, et al. A phase II study of clinical activity of SCH 717454 (robatumumab) in patients with relapsed osteosarcoma and Ewing sarcoma: Anderson et al. Pediatr Blood Cancer. 2016;63(10):1761-1770. doi:10.1002/pbc.26087
106. D’Angelo SP, Shoushtari AN, Keohan ML, et al. Combined KIT and CTLA-4 Blockade in Patients with Refractory GIST and Other Advanced Sarcomas: A Phase Ib Study of Dasatinib plus Ipilimumab. Clin Cancer Res Off J Am Assoc Cancer Res. 2017;23(12):2972-2980. doi:10.1158/1078-0432.CCR-16-2349
107. Chugh R, Griffith KA, Davis EJ, et al. Doxorubicin plus the IGF-1R antibody cixutumumab in soft tissue sarcoma: a phase I study using the TITE-CRM model. Ann Oncol. 2015;26(7):1459-1464. doi:10.1093/annonc/mdv171
108. Wagner LM, Fouladi M, Ahmed A, et al. Phase II Study of Cixutumumab in Combination with Temsirolimus in Pediatric Patients and Young Adults with Recurrent or Refractory Sarcoma: A Report from the Children’s Oncology Group. Pediatr Blood Cancer. 2015;62(3):440-444. doi:10.1002/pbc.25334
109. Pappo AS, Vassal G, Crowley JJ, et al. A phase 2 trial of R1507, a monoclonal antibody to the insulin-like growth factor-1 receptor (IGF-1R), in patients with recurrent or refractory rhabdomyosarcoma, osteosarcoma, synovial sarcoma, and other soft tissue sarcomas: Results of a Sarcoma Alliance for Research Through Collaboration study. Cancer. 120(16):2448-2456. doi:10.1002/cncr.28728
110. Schöffski P, Adkins D, Blay J-Y, et al. An open-label, phase 2 study evaluating the efficacy and safety of the anti-IGF-1R antibody cixutumumab in patients with previously treated advanced or metastatic soft-tissue sarcoma or Ewing family of tumours. Eur J Cancer. 2013;49(15):3219-3228. doi:10.1016/j.ejca.2013.06.010
111. Schwartz GK, Tap WD, Qin L-X, et al. Cixutumumab and temsirolimus for patients with bone and soft-tissue sarcoma: a multicentre, open-label, phase 2 trial. Lancet Oncol. 2013;14(4):371-382. doi:10.1016/S1470-2045(13)70049-4
112. Tap WD, Demetri G, Barnette P, et al. Phase II Study of Ganitumab, a Fully Human Anti–Type-1 Insulin-Like Growth Factor Receptor Antibody, in Patients With Metastatic Ewing Family Tumors or Desmoplastic Small Round Cell Tumors. J Clin Oncol. 2012;30(15):1849-1856. doi:10.1200/JCO.2011.37.2359
113. Naing A, LoRusso P, Fu S, et al. Insulin Growth Factor-Receptor (IGF-1R) Antibody Cixutumumab Combined with the mTOR Inhibitor Temsirolimus in Patients with Refractory Ewing’s Sarcoma Family Tumors. Clin Cancer Res Off J Am Assoc Cancer Res. 2012;18(9). doi:10.1158/1078-0432.CCR-12-0061
114. Ha HT, Griffith KA, Zalupski MM, et al. Phase II Trial of Cetuximab in Patients With Metastatic or Locally Advanced Soft Tissue or Bone Sarcoma: Am J Clin Oncol. 2013;36(1):77-82. doi:10.1097/COC.0b013e31823a4970
115. Malempati S, Weigel B, Ingle AM, et al. Phase I/II Trial and Pharmacokinetic Study of Cixutumumab in Pediatric Patients With Refractory Solid Tumors and Ewing Sarcoma: A Report From the Children’s Oncology Group. J Clin Oncol. 2012;30(3):256-262. doi:10.1200/JCO.2011.37.4355
116. Juergens H, Daw NC, Geoerger B, et al. Preliminary Efficacy of the Anti-Insulin–Like Growth Factor Type 1 Receptor Antibody Figitumumab in Patients With Refractory Ewing Sarcoma. J Clin Oncol. 2011;29(34):4534-4540. doi:10.1200/JCO.2010.33.0670
117. Pappo AS, Patel SR, Crowley J, et al. R1507, a Monoclonal Antibody to the Insulin-Like Growth Factor 1 Receptor, in Patients With Recurrent or Refractory Ewing Sarcoma Family of Tumors: Results of a Phase II Sarcoma Alliance for Research Through Collaboration Study. J Clin Oncol. 2011;29(34):4541-4547. doi:10.1200/JCO.2010.34.0000
118. Quek R, Wang Q, Morgan JA, et al. Combination mTOR and IGF-1R Inhibition: Phase I Trial of Everolimus and Figitumumab in Patients with Advanced Sarcomas and Other Solid Tumors. Clin Cancer Res. 2011;17(4):871-879. doi:10.1158/1078-0432.CCR-10-2621
119. Olmos D, Postel-Vinay S, Molife LR, et al. Safety, pharmacokinetics, and preliminary activity of the anti-IGF-1R antibody figitumumab (CP-751,871) in patients with sarcoma and Ewing’s sarcoma: a phase 1 expansion cohort study. Lancet Oncol. 2010;11(2):129-135. doi:10.1016/S1470-2045(09)70354-7
120. Choi BS, Sondel PM, Hank JA, et al. Phase I trial of combined treatment with ch14.18 and R24 monoclonal antibodies and interleukin-2 for patients with melanoma or sarcoma. Cancer Immunol Immunother. 2006;55(7):761-774. doi:10.1007/s00262-005-0069-7
121. Ebb D, Meyers P, Grier H, et al. Phase II Trial of Trastuzumab in Combination With Cytotoxic Chemotherapy for Treatment of Metastatic Osteosarcoma With Human Epidermal Growth Factor Receptor 2 Overexpression: A Report From the Children’s Oncology Group. J Clin Oncol. 2012;30(20):2545-2551. doi:10.1200/JCO.2011.37.4546
122. D’Adamo DR, Anderson SE, Albritton K, et al. Phase II Study of Doxorubicin and Bevacizumab for Patients With Metastatic Soft-Tissue Sarcomas. J Clin Oncol. 2005;23(28):7135-7142. doi:10.1200/JCO.2005.16.139
123. Ray-Coquard IL, Domont J, Tresch-Bruneel E, et al. Paclitaxel Given Once Per Week With or Without Bevacizumab in Patients With Advanced Angiosarcoma: A Randomized Phase II Trial. J Clin Oncol. 2015;33(25):2797-2802. doi:10.1200/JCO.2015.60.8505
124. Agulnik M, Yarber JL, Okuno SH, et al. An open-label, multicenter, phase II study of bevacizumab for the treatment of angiosarcoma and epithelioid hemangioendotheliomas. Ann Oncol. 2013;24(1):257-263. doi:10.1093/annonc/mds237
125. Monga V, Swami U, Tanas M, et al. A Phase I/II Study Targeting Angiogenesis Using Bevacizumab Combined with Chemotherapy and a Histone Deacetylase Inhibitor (Valproic Acid) in Advanced Sarcomas. Cancers. 2018;10(2):53. doi:10.3390/cancers10020053
126. D’Angelo SP, Mahoney MR, Van Tine BA, et al. Nivolumab with or without ipilimumab treatment for metastatic sarcoma (Alliance A091401): two open-label, non-comparative, randomised, phase 2 trials. Lancet Oncol. 2018;19(3):416-426. doi:10.1016/S1470-2045(18)30006-8
127. Gupta S, Gouw L, Wright J, et al. Phase II study of amrubicin (SM-5887), a synthetic 9-aminoanthracycline, as first line treatment in patients with metastatic or unresectable soft tissue sarcoma: durable response in myxoid liposarcoma with TLS-CHOP translocation. Invest New Drugs. 2016;34(2):243-252. doi:10.1007/s10637-016-0333-z
128. Sanctis RD, Bertuzzi A, Basso U, et al. Non-pegylated Liposomal Doxorubicin plus Ifosfamide in Metastatic Soft Tissue Sarcoma: Results from a Phase-II Trial. Anticancer Res. 2015;35(1):543-547.
129. Stroppa E, Bertuzzi A, Di Comite G, et al. Phase I study of non-pegylated liposomal doxorubicin in combination with ifosfamide in adult patients with metastatic soft tissue sarcomas. Invest New Drugs. 2010;28(6):834-838. doi:10.1007/s10637-009-9288-7
130. Nielsen OS, Reichardt P, Christensen TB, et al. Phase 1 European Organisation for Research and Treatment of Cancer study determining safety of pegylated liposomal doxorubicin (Caelyx®) in combination with ifosfamide in previously untreated adult patients with advanced or metastatic soft tissue sarcomas. Eur J Cancer. 2006;42(14):2303-2309. doi:10.1016/j.ejca.2006.04.011
131. Sutton G, Blessing J, Hanjani P, Kramer P. Phase II evaluation of liposomal doxorubicin (Doxil) in recurrent or advanced leiomyosarcoma of the uterus: a Gynecologic Oncology Group study. Gynecol Oncol. 2005;96(3):749-752. doi:10.1016/j.ygyno.2004.11.036
132. Toma S, Tucci A, Villani G, Carteni G, Spadini N, Palumbo R. Liposomal doxorubicin (Caelyx) in advanced pretreated soft tissue sarcomas: a phase II study of the Italian Sarcoma Group (ISG). Anticancer Res. 2000;20(1B):485-491.
133. Harter P, Sehouli J, Reuss A, et al. Phase II Study Evaluating PegLiposomal Doxorubicin and Carboplatin Combination Chemotherapy in Gynecologic Sarcomas and Mixed Epithelial-Mesenchymal Tumors A Phase II Protocol of the Arbeitsgemeinschaft Gynaekologische Onkologie Study Group (AGO-GYN 7): Int J Gynecol Cancer. 2016;26(9):1636-1641. doi:10.1097/IGC.0000000000000831
134. Bafaloukos D, Papadimitriou C, Linardou H, et al. Combination of pegylated liposomal doxorubicin (PLD) and paclitaxel in patients with advanced soft tissue sarcoma: a phase II study of the Hellenic Cooperative Oncology Group. Br J Cancer. 2004;91(9):1639-1644. doi:10.1038/sj.bjc.6602148
135. Skubitz KM. Phase II Trial Of Pegylated-liposomal Doxorubicin (DoxilTM) In Sarcoma*. Cancer Invest. 2003;21(2):167-176. doi:10.1081/CNV-120016412
136. Judson I, Radford JA, Harris M. Randomised phase II trial of pegylated liposomal doxorubicin (DOXIL1/CAELYX1) versus doxorubicin in the treatment of advanced or metastatic soft tissue sarcoma: a study by the EORTC Soft Tissue and Bone Sarcoma Group. Eur J Cancer. 2001:8.
137. Chidiac T, Budd GT, Pelley R, et al. Phase II trial of liposomal doxorubicin (Doxil) in advanced soft tissue sarcomas. Invest New Drugs. 2000;18(3):253-259.
138. Nooij MA, Whelan J, Bramwell VHC, et al. Doxorubicin and cisplatin chemotherapy in high-grade spindle cell sarcomas of the bone, other than osteosarcoma or malignant fibrous histiocytoma: a European Osteosarcoma Intergroup Study. Eur J Cancer. 2005;41(2):225-230. doi:10.1016/j.ejca.2004.08.026
139. Phase 1/2 study of immunotherapy with dendritic cells pulsed with autologous tumor lysate in patients with refractory bone and soft tissue sarcoma - Miwa - 2017 - Cancer - Wiley Online Library. https://proxy.library.upenn.edu:2894/doi/full/10.1002/cncr.30606. Accessed July 9, 2018.
140. Ahmed N, Brawley VS, Hegde M, et al. Human Epidermal Growth Factor Receptor 2 (HER2) –Specific Chimeric Antigen Receptor–Modified T Cells for the Immunotherapy of HER2-Positive Sarcoma. J Clin Oncol. 2015;33(15):1688-1696. doi:10.1200/JCO.2014.58.0225
141. Mahvi DM, Shi F-S, Yang N-S, et al. Immunization by Particle-Mediated Transfer of the Granulocyte-Macrophage Colony-Stimulating Factor Gene into Autologous Tumor Cells in Melanoma or Sarcoma Patients: Report of a Phase I/IB Study. Hum Gene Ther. 2002;13(14):1711-1721. doi:10.1089/104303402760293556
142. Gravis G, Mousseau M, Douillard JY, et al. Can interleukin-2 reverse anthracyclin chemoresistance in metastatic soft tissue sarcoma patients. Results of a prospective phase II clinical trial. Eur Cytokine Netw. 2001;12(2):239-243.
143. Krishnadas DK, Shusterman S, Bai F, et al. A phase I trial combining decitabine/dendritic cell vaccine targeting MAGE-A1, MAGE-A3 and NY-ESO-1 for children with relapsed or therapy-refractory neuroblastoma and sarcoma. Cancer Immunol Immunother. 2015;64(10):1251-1260. doi:10.1007/s00262-015-1731-3
144. Pritchard-Jones K, Spendlove I, Wilton C, et al. Immune responses to the 105AD7 human anti-idiotypic vaccine after intensive chemotherapy, for osteosarcoma. Br J Cancer. 2005;92(8):1358-1365. doi:10.1038/sj.bjc.6602500
145. Dillman R, Barth N, Selvan S, et al. Phase I/II trial of autologous tumor cell line-derived vaccines for recurrent or metastatic sarcomas. Cancer Biother Radiopharm. 2004;19(5):581-588. doi:10.1089/cbr.2004.19.581
146. Goldberg J, Fisher DE, Demetri GD, et al. Biologic Activity of Autologous, Granulocyte-Macrophage Colony Stimulating Factor Secreting Alveolar Soft Parts Sarcoma and Clear Cell Sarcoma Vaccines. Clin Cancer Res Off J Am Assoc Cancer Res. 2015;21(14):3178-3186. doi:10.1158/1078-0432.CCR-14-2932
147. Takahashi R, Ishibashi Y, Hiraoka K, et al. Phase II study of personalized peptide vaccination for refractory bone and soft tissue sarcoma patients. Cancer Sci. 104(10):1285-1294. doi:10.1111/cas.12226
148. Himoudi N, Wallace R, Parsley KL, et al. Lack of T-cell responses following autologous tumour lysate pulsed dendritic cell vaccination, in patients with relapsed osteosarcoma. Clin Transl Oncol. 2012;14(4):271-279. doi:10.1007/s12094-012-0795-1
149. Ullenhag GJ, Spendlove I, Watson NFS, Kallmeyer C, Pritchard-Jones K, Durrant LG. T-cell responses in osteosarcoma patients vaccinated with an anti-idiotypic antibody, 105AD7, mimicking CD55. Clin Immunol. 2008;128(2):148-154. doi:10.1016/j.clim.2008.03.512
150. Edmonson JH, Blessing JA, Cosin JA, Miller DS, Cohn DE, Rotmensch J. Phase II Study of Mitomycin, Doxorubicin, and Cisplatin in the Treatment of Advanced Uterine Leiomyosarcoma: A Gynecologic Oncology Group Study. Gynecol Oncol. 2002;85(3):507-510. doi:10.1006/gyno.2002.6661
151. Morland B, Platt K, Whelan JS. A phase II window study of irinotecan (CPT-11) in high risk ewing sarcoma: A Euro-E.W.I.N.G. study. Pediatr Blood Cancer. 61(3):442-445. doi:10.1002/pbc.24767
152. Bochennek K, Dantonello T, Koscielniak E, et al. Response of Children with Stage IV Soft Tissue Sarcoma to Topotecan and Carboplatin: A Phase II Window Trial of the Cooperative Soft Tissue Sarcoma Group. Klin Pädiatr. 2013;225(06):309-314. doi:10.1055/s-0033-1341489
153. Reichardt P, Nielsen OS, Bauer S, et al. Exatecan in pretreated adult patients with advanced soft tissue sarcoma: Results of a phase II – Study of the EORTC Soft Tissue and Bone Sarcoma Group. Eur J Cancer. 2007;43(6):1017-1022. doi:10.1016/j.ejca.2007.01.014
154. Seibel NL, Krailo M, Chen Z, et al. Upfront window trial of topotecan in previously untreated children and adolescents with poor prognosis metastatic osteosarcoma. Cancer. 109(8):1646-1653. doi:10.1002/cncr.22553
155. Pappo AS, Lyden E, Breitfeld P, et al. Two Consecutive Phase II Window Trials of Irinotecan Alone or in Combination With Vincristine for the Treatment of Metastatic Rhabdomyosarcoma: The Children’s Oncology Group. J Clin Oncol. 2007;25(4):362-369. doi:10.1200/JCO.2006.07.1720
156. Vassal G, Couanet D, Stockdale E, et al. Phase II Trial of Irinotecan in Children With Relapsed or Refractory Rhabdomyosarcoma: A Joint Study of the French Society of Pediatric Oncology and the United Kingdom Children’s Cancer Study Group. J Clin Oncol. 2007;25(4):356-361. doi:10.1200/JCO.2006.06.1960
157. Chugh R, Dunn R, Zalupski MM, et al. Phase II Study of 9-Nitro-Camptothecin in Patients With Advanced Chordoma or Soft Tissue Sarcoma. J Clin Oncol. 2005;23(15):3597-3604. doi:10.1200/JCO.2005.02.170
158. Walterhouse DO, Lyden ER, Breitfeld PP, Qualman SJ, Wharam MD, Meyer WH. Efficacy of Topotecan and Cyclophosphamide Given in a Phase II Window Trial in Children With Newly Diagnosed Metastatic Rhabdomyosarcoma: A Children’s Oncology Group Study. J Clin Oncol. 2004;22(8):1398-1403. doi:10.1200/JCO.2004.05.184
159. Reichardt P, Oechsle K, Pink D, Bokemeyer C, al et. An open label, non-comparative phase II study of topotecan as salvage treatment for patients with soft tissue sarcoma. Investig New Drugs N Y. 2003;21(4):481-486.
160. Patel SR, Beach J, Papadopoulos N, et al. Results of a 2-arm Phase II study of 9-nitrocamptothecin in patients with advanced soft-tissue sarcomas. Cancer. 97(11):2848-2852. doi:10.1002/cncr.11385
161. Pappo AS, Lyden E, Breneman J, et al. Up-Front Window Trial of Topotecan in Previously Untreated Children and Adolescents With Metastatic Rhabdomyosarcoma: An Intergroup Rhabdomyosarcoma Study. J Clin Oncol. 2001;19(1):213-219. doi:10.1200/JCO.2001.19.1.213
162. Owens C, Laurence V, Benboubker L, et al. Phase II study of cisplatin and oral VP16 in patients with refractory or relapsed Ewing sarcoma. Cancer Chemother Pharmacol. 2013;71(2):399-404. doi:10.1007/s00280-012-2015-7
163. Miller DS, Blessing JA, Kilgore LC, Mannel R, Van Le L. Phase II trial of topotecan in patients with advanced, persistent, or recurrent uterine leiomyosarcomas: a Gynecologic Oncology Group Study. Am J Clin Oncol. 2000;23(4):355-357.
164. Miller DS, Blessing JA, Schilder J, Munkarah A, Lee YC. Phase II evaluation of topotecan in carcinosarcoma of the uterus: A Gynecologic Oncology Group study. Gynecol Oncol. 2005;98(2):217-221. doi:10.1016/j.ygyno.2005.05.015
165. Otsuki A, Watanabe Y, Nomura H, et al. Paclitaxel and Carboplatin in Patients With Completely or Optimally Resected Carcinosarcoma of the Uterus: A Phase II Trial by the Japanese Uterine Sarcoma Group and the Tohoku Gynecologic Cancer Unit. Int J Gynecol Cancer. 2015;25(1):92-97. doi:10.1097/IGC.0000000000000302
166. Duska LR, Blessing JA, Rotmensch J, et al. A Phase II Evaluation of Ixabepilone (IND #59699, NSC #710428) in the Treatment of Recurrent or Persistent Leiomyosarcoma of the Uterus: an NRG Oncology/Gynecologic Oncology Group Study. Gynecol Oncol. 2014;135(1):44-48. doi:10.1016/j.ygyno.2014.07.101
167. Takano T, Otsuki T, Tokunaga H, et al. Paclitaxel-carboplatin for advanced or recurrent carcinosarcoma of the uterus: the Japan Uterine Sarcoma Group and Tohoku Gynecologic Cancer Unit Study. Int J Clin Oncol. 2014;19(6):1052-1058. doi:10.1007/s10147-013-0658-y
168. Yoo HJ, Lim MC, Lim S, et al. Phase II study of paclitaxel in combination with carboplatin for patients with recurrent or persistent uterine sarcoma. Arch Gynecol Obstet. 2012;286(6):1529-1535. doi:10.1007/s00404-012-2466-4
169. Minard-Colin V, Ichante J-L, Nguyen L, et al. Phase II study of vinorelbine and continuous low doses cyclophosphamide in children and young adults with a relapsed or refractory malignant solid tumour: Good tolerance profile and efficacy in rhabdomyosarcoma – A report from the Société Française des Cancers et leucémies de l’Enfant et de l’adolescent (SFCE). Eur J Cancer. 2012;48(15):2409-2416. doi:10.1016/j.ejca.2012.04.012
170. Pappo AS, Devidas M, Jenkins J, et al. Phase II Trial of Neoadjuvant Vincristine, Ifosfamide, and Doxorubicin With Granulocyte Colony-Stimulating Factor Support in Children and Adolescents With Advanced-Stage Nonrhabdomyosarcomatous Soft Tissue Sarcomas: A Pediatric Oncology Group Study. J Clin Oncol. 2005;23(18):4031-4038. doi:10.1200/JCO.2005.03.209
171. Gallup DG, Blessing JA, Andersen W, Morgan MA. Evaluation of paclitaxel in previously treated leiomyosarcoma of the uterus: a gynecologic oncology group study. Gynecol Oncol. 2003;89(1):48-51. doi:10.1016/S0090-8258(02)00136-1
172. Pivot X, Chevreau C, Cupissol D, et al. Phase II trial of paclitaxel-epirubicin in patients with recurrent soft-tissue sarcoma. Am J Clin Oncol. 2002;25(6):561-564.
173. Kostler WJ, Brodowicz T, Attems Y, et al. Docetaxel as rescue medication in anthracycline- and ifosfamide-resistant locally advanced or metastatic soft tissue sarcoma: Results of a phase II trial. 2018:8.
174. Patel S, Keohan ML, Saif MW, et al. Phase II study of intravenous TZT-1027 in patients with advanced or metastatic soft-tissue sarcomas with prior exposure to anthracycline-based chemotherapy. Cancer. 107(12):2881-2887. doi:10.1002/cncr.22334
175. Suppiah R, Wood L, Elson P, Budd GT. Phase I/II study of docetaxel, ifosfamide, and doxorubicin in advanced, recurrent, or metastatic soft tissue sarcoma (STS). Invest New Drugs. 2006;24(6):509-514. doi:10.1007/s10637-006-9035-2
176. Okuno S, Maples WJ, Mahoney MR, et al. Evaluation of Epothilone B Analog in Advanced Soft Tissue Sarcoma: A Phase II Study of the Phase II Consortium. J Clin Oncol. 2005;23(13):3069-3073. doi:10.1200/JCO.2005.00.372
177. Verweij J, Lee SM, Ruka W, et al. Randomized Phase II Study of Docetaxel Versus Doxorubicin in First- and Second-Line Chemotherapy for Locally Advanced or Metastatic Soft Tissue Sarcomas in Adults: A Study of the European Organization for Research and Treatment of Cancer Soft Tissue and Bone Sarcoma Group. J Clin Oncol. 2000;18(10):2081-2086. doi:10.1200/JCO.2000.18.10.2081
178. Penel N, Bui BN, Bay J-O, et al. Phase II Trial of Weekly Paclitaxel for Unresectable Angiosarcoma: The ANGIOTAX Study. J Clin Oncol. 2008;26(32):5269-5274. doi:10.1200/JCO.2008.17.3146
179. Lacour RA, Euscher E, Atkinson EN, et al. A Phase II Trial of Paclitaxel and Carboplatin in Women With Advanced or Recurrent Uterine Carcinosarcoma: Int J Gynecol Cancer. 2011;21(3):517-522. doi:10.1097/IGC.0b013e31820da9e2
180. Powell MA, Filiaci VL, Rose PG, et al. Phase II Evaluation of Paclitaxel and Carboplatin in the Treatment of Carcinosarcoma of the Uterus: A Gynecologic Oncology Group Study. J Clin Oncol. 2010;28(16):2727-2731. doi:10.1200/JCO.2009.26.8326
181. Davis EJ, Chugh R, Zhao L, et al. A randomised, open-label, phase II study of neo/adjuvant doxorubicin and ifosfamide versus gemcitabine and docetaxel in patients with localised, high-risk, soft tissue sarcoma. Eur J Cancer. 2015;51(13):1794-1802. doi:10.1016/j.ejca.2015.05.010
182. Martin-Broto J, Redondo A, Valverde C, et al. Gemcitabine plus sirolimus for relapsed and progressing osteosarcoma patients after standard chemotherapy: a multicenter, single-arm phase II trial of Spanish Group for Research on Sarcoma (GEIS). Ann Oncol. 2017;28(12):2994-2999. doi:10.1093/annonc/mdx536
183. Luo Z, Zhang X, Peng W, et al. A Phase II Study of Gemcitabine, Vincristine, and Cisplatin (Gvp) As Second-Line Treatment for Patients with Advanced Soft Tissue Sarcoma: Medicine (Baltimore). 2015;94(43):e1777. doi:10.1097/MD.0000000000001777
184. Munhoz RR, D’Angelo SP, Gounder MM, et al. A Phase Ib/II Study of Gemcitabine and Docetaxel in Combination With Pazopanib for the Neoadjuvant Treatment of Soft Tissue Sarcomas. The Oncologist. 2015;20(11):1245-1246. doi:10.1634/theoncologist.2015-0245
185. Pautier P, Floquet A, Penel N, et al. Randomized Multicenter and Stratified Phase II Study of Gemcitabine Alone Versus Gemcitabine and Docetaxel in Patients with Metastatic or Relapsed Leiomyosarcomas: A Fédération Nationale des Centres de Lutte Contre le Cancer (FNCLCC) French Sarcoma Group Study (TAXOGEM study). The Oncologist. 2012;17(9):1213-1220. doi:10.1634/theoncologist.2011-0467
186. Hartmann JT, Bauer S, Egerer G, et al. Pemetrexed in patients with refractory soft tissue sarcoma: A non-comparative multicenter phase II study of the German Sarcoma Group AIO-STS 005. Invest New Drugs. 2013;31(1):167-174. doi:10.1007/s10637-012-9840-8
187. Fox E, Patel S, Wathen JK, et al. Phase II Study of Sequential Gemcitabine Followed by Docetaxel for Recurrent Ewing Sarcoma, Osteosarcoma, or Unresectable or Locally Recurrent Chondrosarcoma: Results of Sarcoma Alliance for Research Through Collaboration Study 003. The Oncologist. 2012;17(3):321. doi:10.1634/theoncologist.2010-0265
188. Duffaud F, Egerer G, Ferrari S, Rassam H, Boecker U, Bui-Nguyen B. A phase II trial of second-line pemetrexed in adults with advanced/metastatic osteosarcoma. Eur J Cancer. 2012;48(4):564-570. doi:10.1016/j.ejca.2011.12.015
189. García-del-Muro X, López-Pousa A, Maurel J, et al. Randomized Phase II Study Comparing Gemcitabine Plus Dacarbazine Versus Dacarbazine Alone in Patients With Previously Treated Soft Tissue Sarcoma: A Spanish Group for Research on Sarcomas Study. J Clin Oncol. 2011;29(18):2528-2533. doi:10.1200/JCO.2010.33.6107
190. DuBois SG, Krailo MD, Lessnick SL, et al. Phase II Study of Intermediate-Dose Cytarabine in Patients with Relapsed or Refractory Ewing Sarcoma: A Report from the Children’s Oncology Group. Pediatr Blood Cancer. 2009;52(3):324-327. doi:10.1002/pbc.21822
191. Dileo P, Morgan JA, Zahrieh D, et al. Gemcitabine and vinorelbine combination chemotherapy for patients with advanced soft tissue sarcomas. Cancer. 109(9):1863-1869. doi:10.1002/cncr.22609
192. Losa R, Fra J, López-Pousa A, et al. Phase II study with the combination of gemcitabine and DTIC in patients with advanced soft tissue sarcomas. Cancer Chemother Pharmacol. 2007;59(2):251-259. doi:10.1007/s00280-006-0263-0
193. López-Pousa A, Losa R, Martín J, et al. Phase I/II trial of doxorubicin and fixed dose-rate infusion gemcitabine in advanced soft tissue sarcomas: a GEIS study. Br J Cancer. 2006;94(12):1797-1802. doi:10.1038/sj.bjc.6603187
194. Von Burton G, Rankin C, Zalupski MM, Mills GM, Borden EC, Karen A. Phase II Trial of Gemcitabine as First Line Chemotherapy in Patients With Metastatic or Unresectable Soft Tissue Sarcoma: Am J Clin Oncol. 2006;29(1):59-61. doi:10.1097/01.coc.0000195088.28956.dd
195. Hartmann JT, Oechsle K, Huober J, et al. An open label, non-comparative phase II study of gemcitabine as salvage treatment for patients with pretreated adult type soft tissue sarcoma. Invest New Drugs. 2006;24(3):249-253. doi:10.1007/s10637-005-3537-1
196. Look KY, Sandler A, Blessing JA, Lucci JA, Rose PG, Gynecologic Oncology Group (GOG) Study. Phase II trial of gemcitabine as second-line chemotherapy of uterine leiomyosarcoma: a Gynecologic Oncology Group (GOG) Study. Gynecol Oncol. 2004;92(2):644-647. doi:10.1016/j.ygyno.2003.11.023
197. Okuno S, Ryan LM, Edmonson JH, Priebat DA, Blum RH. Phase II trial of gemcitabine in patients with advanced sarcomas (E1797). Cancer. 97(8):1969-1973. doi:10.1002/cncr.11290
198. Okuno S, Edmonson J, Mahoney M, Buckner JC, Frytak S, Galanis E. Phase II trial of gemcitabine in advanced sarcomas. Cancer. 2002;94(12):3225-3229. doi:10.1002/cncr.10602
199. Patel SR, Gandhi V, Jenkins J, et al. Phase II Clinical Investigation of Gemcitabine in Advanced Soft Tissue Sarcomas and Window Evaluation of Dose Rate on Gemcitabine Triphosphate Accumulation. J Clin Oncol. 2001;19(15):3483-3489. doi:10.1200/JCO.2001.19.15.3483
200. Phase II trial of gemcitabine in patients with pretreated advanced soft tissue sarcomas. https://proxy.library.upenn.edu:3083/pubmed?pmid=10912948. Accessed July 26, 2018.
201. Svancárová L, Blay JY, Judson IR, et al. Gemcitabine in advanced adult soft-tissue sarcomas. A phase II study of the EORTC Soft Tissue and Bone Sarcoma Group. Eur J Cancer Oxf Engl 1990. 2002;38(4):556-559.
202. Hensley ML, Blessing JA, Mannel R, Rose PG. Fixed-dose rate gemcitabine plus docetaxel as first-line therapy for metastatic uterine leiomyosarcoma: a Gynecologic Oncology Group phase II trial. Gynecol Oncol. 2008;109(3):329-334. doi:10.1016/j.ygyno.2008.03.010
203. Hensley ML, Blessing JA, DeGeest K, Abulafia O, Rose PG, Homesley HD. Fixed-dose rate gemcitabine plus docetaxel as second-line therapy for metastatic uterine leiomyosarcoma: a Gynecologic Oncology Group phase II study. Gynecol Oncol. 2008;109(3):323-328. doi:10.1016/j.ygyno.2008.02.024
204. Hensley ML, Maki R, Venkatraman E, et al. Gemcitabine and Docetaxel in Patients With Unresectable Leiomyosarcoma: Results of a Phase II Trial. J Clin Oncol. 2002;20(12):2824-2831. doi:10.1200/JCO.2002.11.050
205. Martin-Liberal J, López-Pousa A, Martínez-Trufero J, et al. Phase II Study of Gemcitabine Plus Sirolimus in Previously Treated Patients with Advanced Soft-Tissue Sarcoma: a Spanish Group for Research on Sarcomas (GEIS) Study. Target Oncol. 2018;13(1):81-87. doi:10.1007/s11523-017-0539-9
206. Buesa JM, Losa R, Fernández A, et al. Phase I clinical trial of fixed–dose rate infusional gemcitabine and dacarbazine in the treatment of advanced soft tissue sarcoma, with assessment of gemcitabine triphosphate accumulation. Cancer. 101(10):2261-2269. doi:10.1002/cncr.20612
207. Schmitt T, Mayer-Steinacker R, Mayer F, et al. Vorinostat in refractory soft tissue sarcomas – Results of a multi-centre phase II trial of the German Soft Tissue Sarcoma and Bone Tumour Working Group (AIO). Eur J Cancer. 2016;64:74-82. doi:10.1016/j.ejca.2016.05.018
208. Thomas S, Aggarwal R, Jahan T, et al. A phase I trial of panobinostat and epirubicin in solid tumors with a dose expansion in patients with sarcoma. Ann Oncol. 2016;27(5):947-952. doi:10.1093/annonc/mdw044
209. Chu QS-C, Nielsen TO, Alcindor T, et al. A phase II study of SB939, a novel pan-histone deacetylase inhibitor, in patients with translocation-associated recurrent/metastatic sarcomas—NCIC-CTG IND 200. Ann Oncol. 2015;26(5):973-981. doi:10.1093/annonc/mdv033
210. Choy E, Flamand Y, Balasubramanian S, et al. Phase I Study of Oral Abexinostat, a Histone Deacetylase Inhibitor, in Combination with Doxorubicin in Patients with Metastatic Sarcoma. Cancer. 2015;121(8):1223-1230. doi:10.1002/cncr.29175
211. Cassier PA, Lefranc A, Y Amela E, et al. A phase II trial of panobinostat in patients with advanced pretreated soft tissue sarcoma. A study from the French Sarcoma Group. Br J Cancer. 2013;109(4):909-914. doi:10.1038/bjc.2013.442
212. Gounder MM, Zer A, Tap WD, et al. Phase IB Study of Selinexor, a First-in-Class Inhibitor of Nuclear Export, in Patients With Advanced Refractory Bone or Soft Tissue Sarcoma. J Clin Oncol. 2016;34(26):3166-3174. doi:10.1200/JCO.2016.67.6346
213. Toulmonde M, Le Cesne A, Piperno-Neumann S, et al. Aplidin in patients with advanced dedifferentiated liposarcomas: a French Sarcoma Group Single-Arm Phase II study. Ann Oncol. 2015;26(7):1465-1470. doi:10.1093/annonc/mdv195
214. Choy E, Butrynski JE, Harmon DC, et al. Phase II study of olaparib in patients with refractory Ewing sarcoma following failure of standard chemotherapy. BMC Cancer. 2014;14(1). doi:10.1186/1471-2407-14-813
215. Jones RL, Ferrari S, Blay JY, et al. A Phase II multicenter, open-label, clinical and pharmokinetic trial of PM00104 in patients with advanced Ewing Family of Tumors. Invest New Drugs. 2014;32(1):171-177. doi:10.1007/s10637-013-0037-6
216. Italiano A, Le Cesne A, Bellera C, et al. GDC-0449 in patients with advanced chondrosarcomas: a French Sarcoma Group/US and French National Cancer Institute Single-Arm Phase II Collaborative Study. Ann Oncol. 2013;24(11):2922-2926. doi:10.1093/annonc/mdt391
217. Wagner AJ, Chugh R, Rosen LS, et al. A phase 1 study of the heat shock protein 90 inhibitor retaspimycin hydrochloride (IPI-504) in patients with gastrointestinal stromal tumors or soft tissue sarcomas. Clin Cancer Res Off J Am Assoc Cancer Res. 2013;19(21):6020-6029. doi:10.1158/1078-0432.CCR-13-0953
218. Chawla SP, Staddon A, Hendifar A, Messam CA, Patwardhan R, Kamel YM. Results of a phase I dose escalation study of eltrombopag in patients with advanced soft tissue sarcoma receiving doxorubicin and ifosfamide. BMC Cancer. 2013;13:121. doi:10.1186/1471-2407-13-121
219. Poklepovic A, Youseffian L, Winning M, et al. Phase I trial of bortezomib and dacarbazine in melanoma and soft tissue sarcoma. Invest New Drugs. 2013;31(4). doi:10.1007/s10637-012-9913-8
220. Pan J, Mott M, Xi B, et al. Phase I study of nelfinavir in liposarcoma. Cancer Chemother Pharmacol. 2012;70(6):791-799. doi:10.1007/s00280-012-1961-4
221. Ryan CW, Matias C, Agulnik M, et al. A phase II study of tasisulam sodium (LY573636 sodium) as second-line or third-line treatment for patients with unresectable or metastatic soft tissue sarcoma. Invest New Drugs. 2013;31(1):145-151. doi:10.1007/s10637-012-9819-5
222. Mackay H, Buckanovich R, Hirte H, et al. A phase II study single agent of aflibercept (VEGF Trap) in patients with recurrent or metastatic gynecologic carcinosarcomas and uterine leiomyosarcoma. A trial of the Princess Margaret Hospital, Chicago and California Cancer Phase II Consortia. Gynecol Oncol. 2012;125(1):136-140. doi:10.1016/j.ygyno.2011.11.042
223. Ganjoo KN, Cranmer LD, Butrynski JE, et al. A Phase I Study of the Safety and Pharmacokinetics of the Hypoxia-Activated Prodrug TH-302 in Combination with Doxorubicin in Patients with Advanced Soft Tissue Sarcoma. Oncology. 2011;80(1-2):50-56. doi:10.1159/000327739
224. Chawla SP, Chua VS, Fernandez L, et al. Phase I/II and Phase II Studies of Targeted Gene Delivery In Vivo: Intravenous Rexin-G for Chemotherapy-resistant Sarcoma and Osteosarcoma. Mol Ther J Am Soc Gene Ther. 2009;17(9):1651-1657. doi:10.1038/mt.2009.126
225. Ramondetta LM, Johnson AJ, Sun CC, et al. Phase 2 trial of mifepristone (RU-486) in advanced or recurrent endometrioid adenocarcinoma or low-grade endometrial stromal sarcoma. Cancer. 115(9):1867-1874. doi:10.1002/cncr.24197
226. Baker LH, Rowinsky EK, Mendelson D, et al. Randomized, Phase II Study of the Thrombospondin-1-Mimetic Angiogenesis Inhibitor ABT-510 in Patients With Advanced Soft Tissue Sarcoma. J Clin Oncol. 2008;26(34):5583-5588. doi:10.1200/JCO.2008.17.4706
227. Ray-Coquard I, Cesne AL, Whelan JS, et al. A Phase II Study of Gefitinib for Patients with Advanced HER-1 Expressing Synovial Sarcoma Refractory to Doxorubicin-Containing Regimens. The Oncologist. 2008;13(4):467-473. doi:10.1634/theoncologist.2008-0065
228. McMeekin DS, Sill MW, Darcy KM, et al. A phase II trial of thalidomide in patients with refractory leiomyosarcoma of the uterus and correlation with biomarkers of angiogenesis: A gynecologic oncology group study. Gynecol Oncol. 2007;106(3):596-603. doi:10.1016/j.ygyno.2007.05.013
229. Chu QSC, Forouzesh B, Syed S, et al. A phase II and pharmacological study of the matrix metalloproteinase inhibitor (MMPI) COL-3 in patients with advanced soft tissue sarcomas. Invest New Drugs. 2007;25(4):359-367. doi:10.1007/s10637-006-9031-6
230. Ryan CW, Dolan ME, Brockstein BB, et al. A phase II trial of O 6-benzylguanine and carmustine in patients with advanced soft tissue sarcoma. Cancer Chemother Pharmacol. 2006;58(5):634-639. doi:10.1007/s00280-006-0210-0
231. Yi-Shin Kuo D, Timmins P, Blank SV, et al. Phase II trial of thalidomide for advanced and recurrent gynecologic sarcoma: A brief communication from the New York Phase II consortium. Gynecol Oncol. 2006;100(1):160-165. doi:10.1016/j.ygyno.2005.08.033
232. Galanis E, Okuno SH, Nascimento AG, et al. Phase I–II trial of ONYX-015 in combination with MAP chemotherapy in patients with advanced sarcomas. Gene Ther. 2005;12(5):437-445. doi:10.1038/sj.gt.3302436
233. Mansky PJ, Liewehr DJ, Steinberg SM, et al. Treatment of metastatic osteosarcoma with the somatostatin analog OncoLar: significant reduction of insulin-like growth factor-1 serum levels. J Pediatr Hematol Oncol. 2002;24(6):440-446.
234. Bramwell VHC, Morris D, Ernst DS, et al. Safety and Efficacy of the Multidrug-Resistance Inhibitor Biricodar (VX-710) with Concurrent Doxorubicin in Patients with Anthracycline-resistant Advanced Soft Tissue Sarcoma. :12.
235. Smith HO, Blessing JA, Vaccarello L. Trimetrexate in the Treatment of Recurrent or Advanced Leiomyosarcoma of the Uterus: A Phase II Study of the Gynecologic Oncology Group. Gynecol Oncol. 2002;84(1):140-144. doi:10.1006/gyno.2001.6482
236. D’ Angelo SP, Mahoney MR, Van Tine BA, et al. Alliance A091103 a phase II study of the angiopoietin 1 and 2 peptibody trebananib for the treatment of angiosarcoma. Cancer Chemother Pharmacol. 2015;75(3):629-638. doi:10.1007/s00280-015-2689-8
237. Maki RG, Kraft AS, Scheu K, et al. A multicenter Phase II study of bortezomib in recurrent or metastatic sarcomas. Cancer. 2005;103(7):1431-1438. doi:10.1002/cncr.20968
238. McMeekin DS, Sill MW, Darcy KM, et al. A phase II trial of thalidomide in patients with refractory uterine carcinosarcoma and correlation with biomarkers of angiogenesis: A Gynecologic Oncology Group study. Gynecol Oncol. 2012;127(2):356-361. doi:10.1016/j.ygyno.2012.07.095
